# Comprehensive Review of Phytochemical Constituents, Pharmacological Properties, and Clinical Applications of *Prunus mume*

**DOI:** 10.3389/fphar.2021.679378

**Published:** 2021-05-28

**Authors:** Xue-Peng Gong, Ying Tang, Yuan-Yuan Song, Guang Du, Juan Li

**Affiliations:** ^1^ Department of Pharmacy, Tongji Hospital, Tongji Medical College, Huazhong University of Science and Technology, Wuhan, China; ^2^ Department of Pharmacy, General Hospital of Central Theater Command, Wuhan, China

**Keywords:** Prunus mume, pharmacological activities, clinical application, functional mechanism, phytochemical constituents

## Abstract

*Prunus mume* is one of the most ancient medicinal herbs and health foods commonly used in Asian countries. It is widely used as a constituent of many medicinal preparations and as a food ingredient for its beneficial health effects. In this review, we retrieved reports from PubMed, embase, Scopus, and SciFinder databases, to collect extensive scientific evidence on the phytochemical constituents, pharmacological properties, and clinical applications of *Prunus mume*. The literature review revealed that approximately 192 compounds have been isolated from different parts of the plant, and their molecular structures have been identified. The pharmacological properties of the plant, including anti-diabetic, liver-protective, antitumor, antimicrobial, antioxidant, and anti-inflammatory activities, as well as their underlying mechanisms, have been clarified by *in vitro* and *in vivo* studies. Clinical studies, although very limited, have been highlighted in this review to provide a reference for further exploration on therapeutic applications of the plant.

## Introduction

*Prunus mume* (Siebold) Siebold&Zucc (*P. mume*) (=*Armeniaca mume*) is an Asian plum species belonging to the Rosaceae family. It is known as *wu mei* (Chinese:乌梅) in China, Japanese apricot or *ume* in Japan, and *maesil* or *oumae* in Korea. The plant is commonly cultivated throughout most of China, and is native to Japan and Korea. The fruit of the plant has been used as a medicinal herb and health food in East Asian countries for more than 2000°years. In China, the dried fruit of *P. mume* is listed in the earliest pharmacopoeia of traditional Chinese medicine (TCM), Shen Nong Ben Cao Jing, compiled during the Han Dynasty, in approximately AD 220. In Japan, the earliest record is found in a medical monograph called *Ishinho* (published in AD 984). According to the Chinese pharmacopoeia records, the dried ripening fruit of *P. mume* can be taken to relieve various physical disorders, such as chronic cough caused by lung deficiency, chronic infectious diarrhea, vomiting, or abdominal pain caused by *Ascaris* infection, dysfunctional uterine bleeding, inadequate secretion of saliva or body fluids. It is also used as a component of many formulas, such as *wu mei wan* (first recorded in *Shang Han Za Bing Lun*, compiled during AD200–210), *Er Chen Tang* and *Chang Shan Yin* (recorded in *Tai Ping Hui Min He Ji Ju Fang*, compiled during AD 1078–1085) to treat different kinds of diseases based on classical theories of TCM. As a common commercial food product, the fruit of *P. mume* is used to prepare pickled plums, plum sauce, plum juice, and plum liquor, which can be consumed as a snack, condiment, or beverage. To date, phytochemical studies have discovered numerous chemical components of the plant, mainly phenolics (Xia et al., 2011; Mitani et al., 2013), flavonoids (Yan et al., 2014a), and organic acids (Gao, 2012). Modern pharmacological studies have disclosed various biological activities and bioactive mechanisms of *P. mume* and its formulas, including antidiabetic (Kishida et al., 2013; Ko et al., 2013), hepatoprotective (Hokari, 2012; Beretta et al., 2016), antitumoral (Hattori et al., 2013; Cho et al., 2019), anti-inflammatory (Morimoto et al., 2009; Mitani et al., 2013), and antimicrobial (Lee and Stein, 2011; Seneviratne et al., 2011) activities. Bailly (2020) reviewed anticancer properties of *P. mume* extracts, however, no comprehensive review on the phytochemical and pharmacological properties of *P. mume* is available.

Herein, we conducted a comprehensive and systematic review to summarize the scattered studies on the phytochemical and pharmacological properties and the clinical applications of *P. mume* to provide a scientific basis for future research directions and better utilization of the plant.

### Data Sources and Search Strategies

A comprehensive literature search was conducted through electronic databases, including PubMed, embase, Scopus, and SciFinder databases. The search time interval was from database inception to 31, Mar 2020. The search strategy used for the PubMed database was “Prunus mume” [tiab] or “Fructus mume” [tiab] or “Chinese plum” [tiab] or “Japanese apricot” [tiab] or “Asian plum” [tiab] or “Oriental plum” [tiab] or “MK 615” [tiab] or maesil [tiab] or oumae [tiab] or ume [tiab] or“Armeniaca mume” [tiab]. The search was limited to English-language and Chinese publications. A PubMed alert was set up for new relevant results.

Database searches initially identified 361 records from Pubmed, 273 records from embase, 540 records from Scopus, and 282 records from the SciFinder database. The duplicate references were removed by Endnote. Only the studies focusing on the phytochemical constituents, pharmacological properties, and clinical applications were selected by two independent reviewers.

### Phytochemical Constituents

**FIGURE 1:**
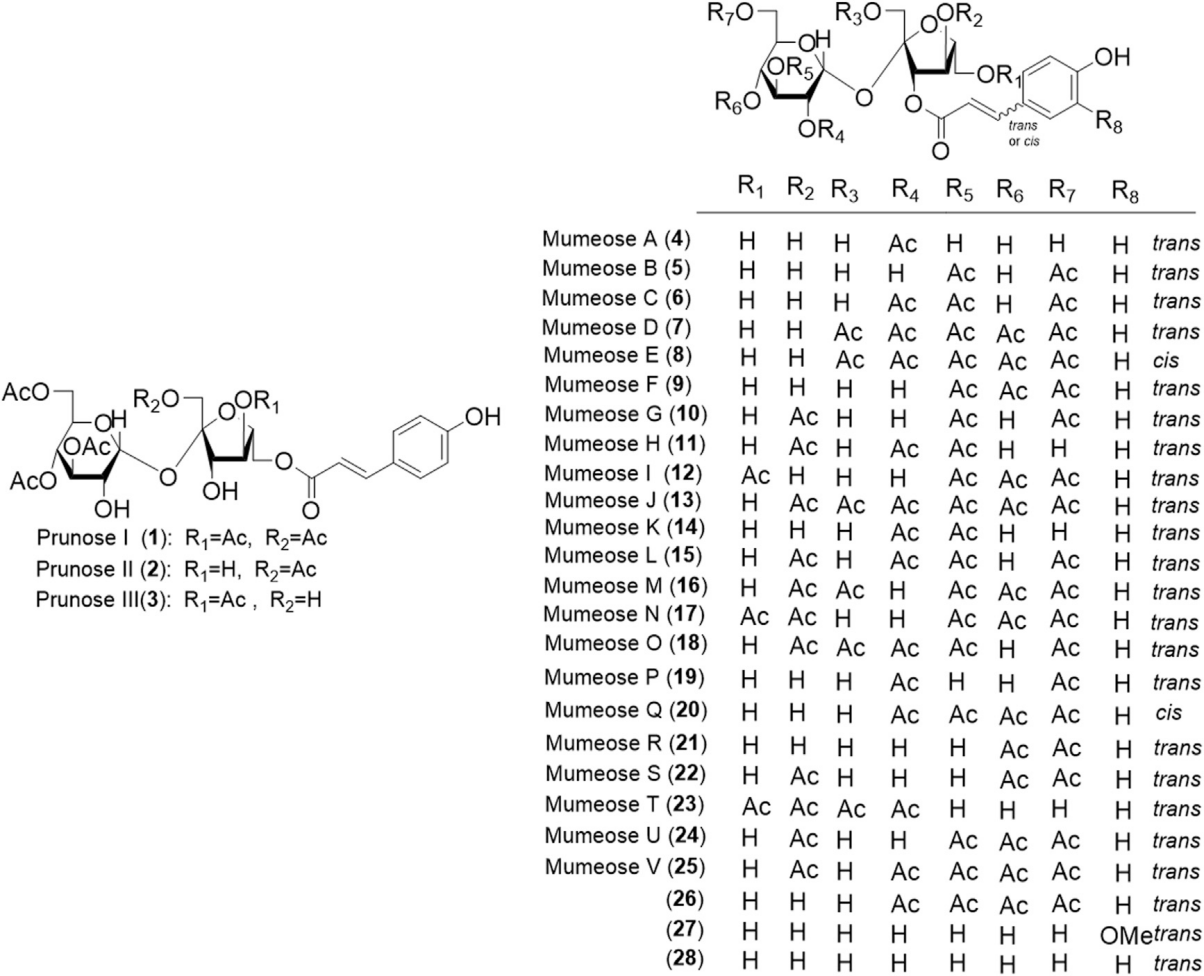
Structures of the phenylpropanoid sucrose esters (1–28) form *P. mume*.

**FIGURE 2:**
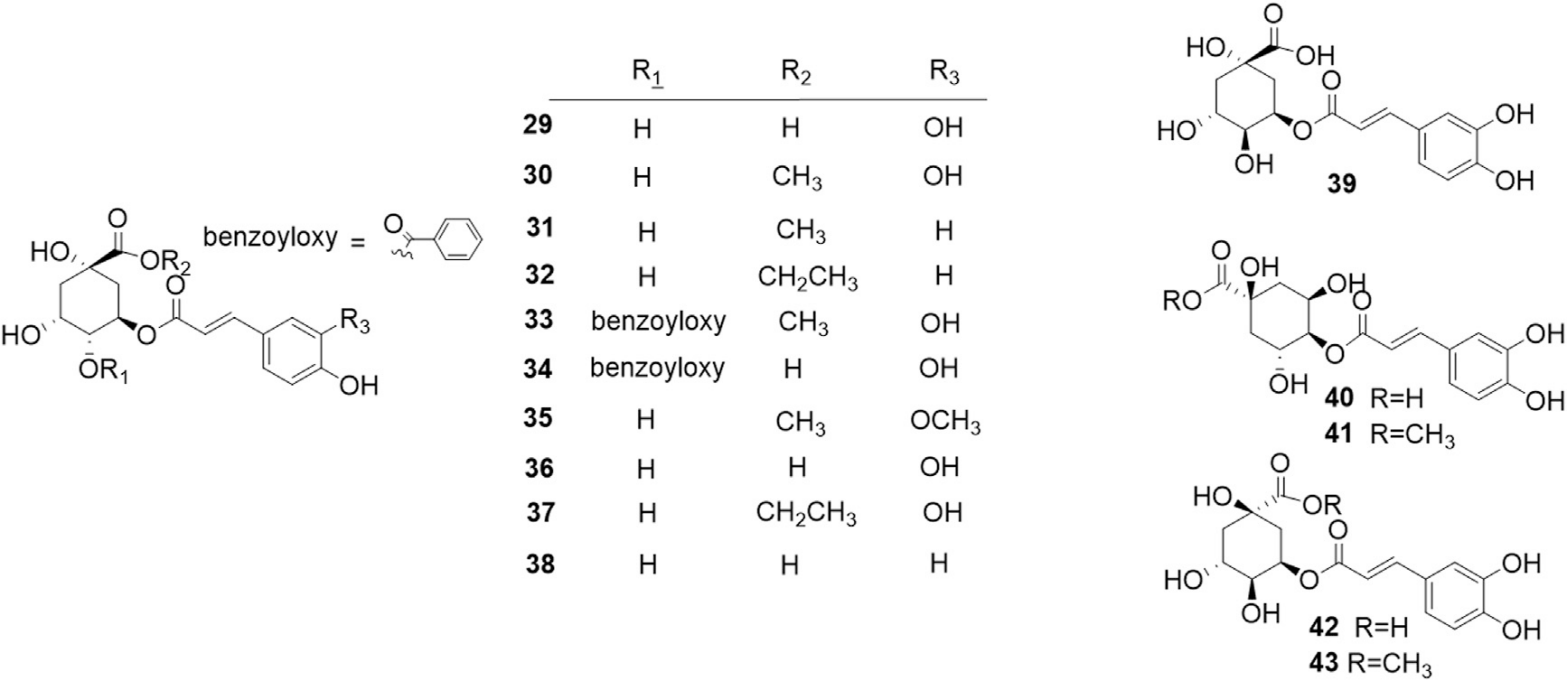
Structures of the hydroxycinnamoylquinic acid derivatives (29–43) form *P. mume*.

**FIGURE 3:**
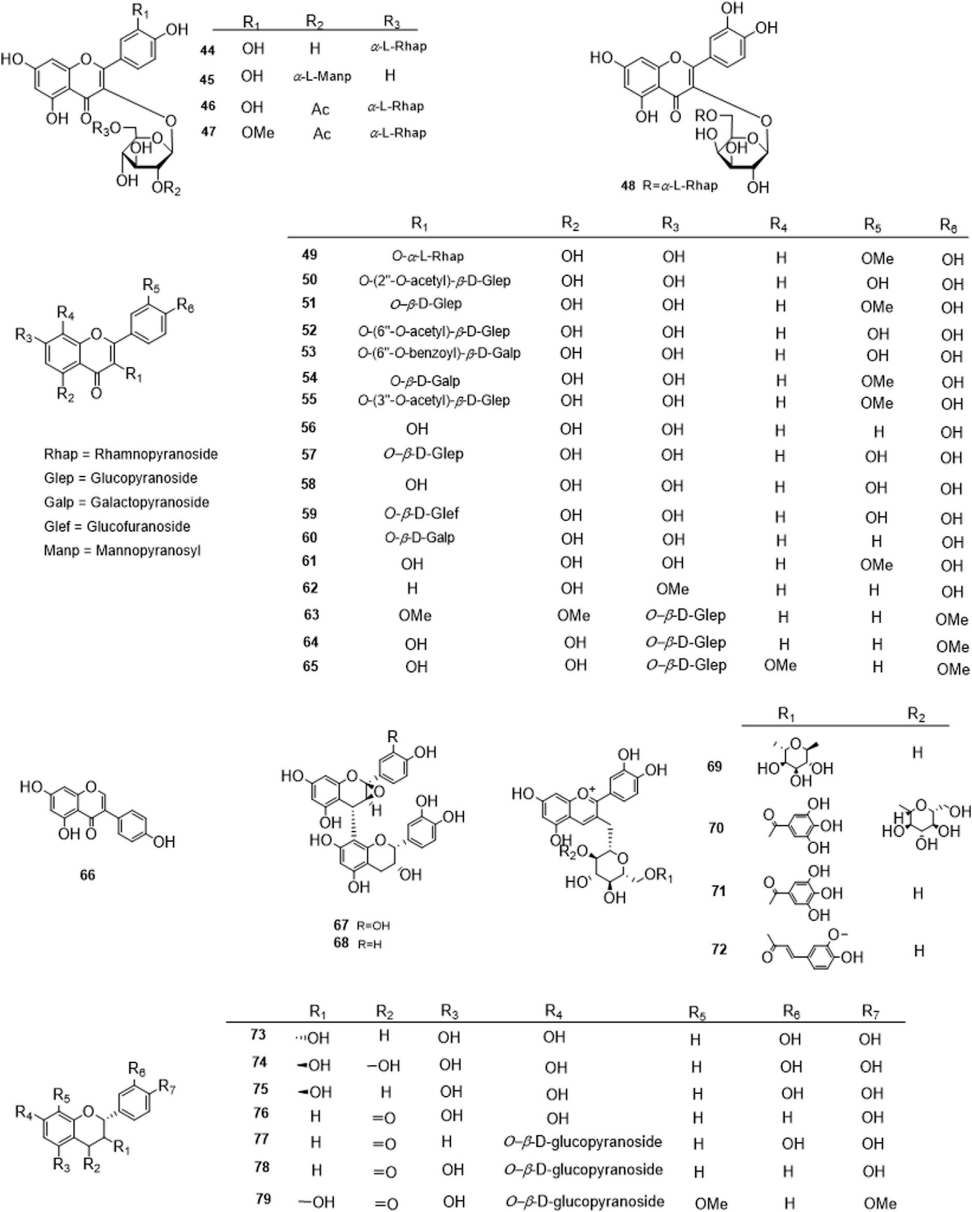
Structures of the flavonoids acid derivatives (44–79) form *P. mume*.

**FIGURE 4:**
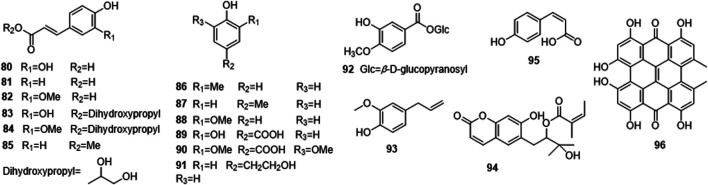
Structures of other phenolics (80–96) form *P. mume*.

**FIGURE 5:**
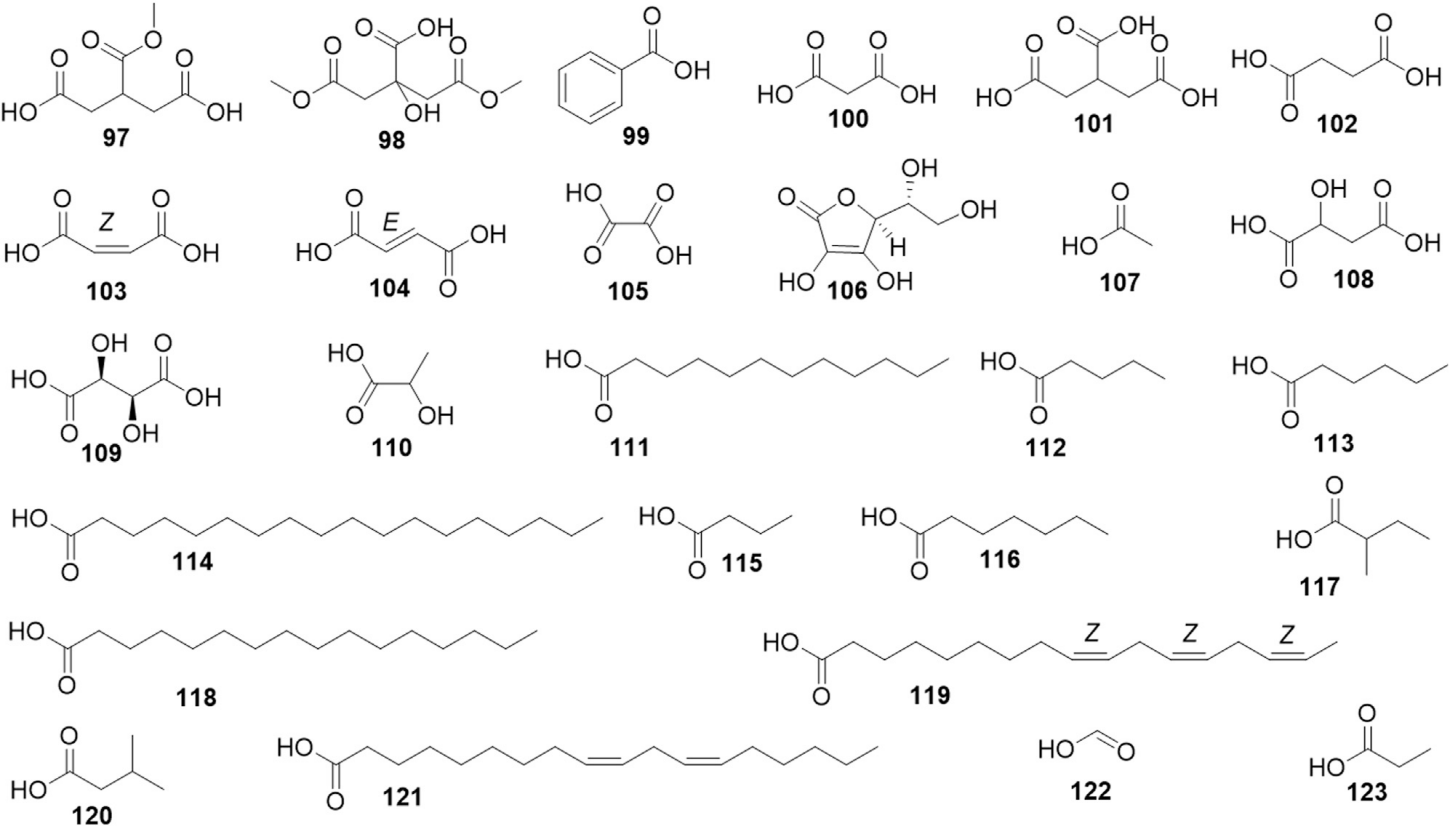
Structures of the Organic acid derivatives (97–123) form *P. mume*.

**FIGURE 6:**
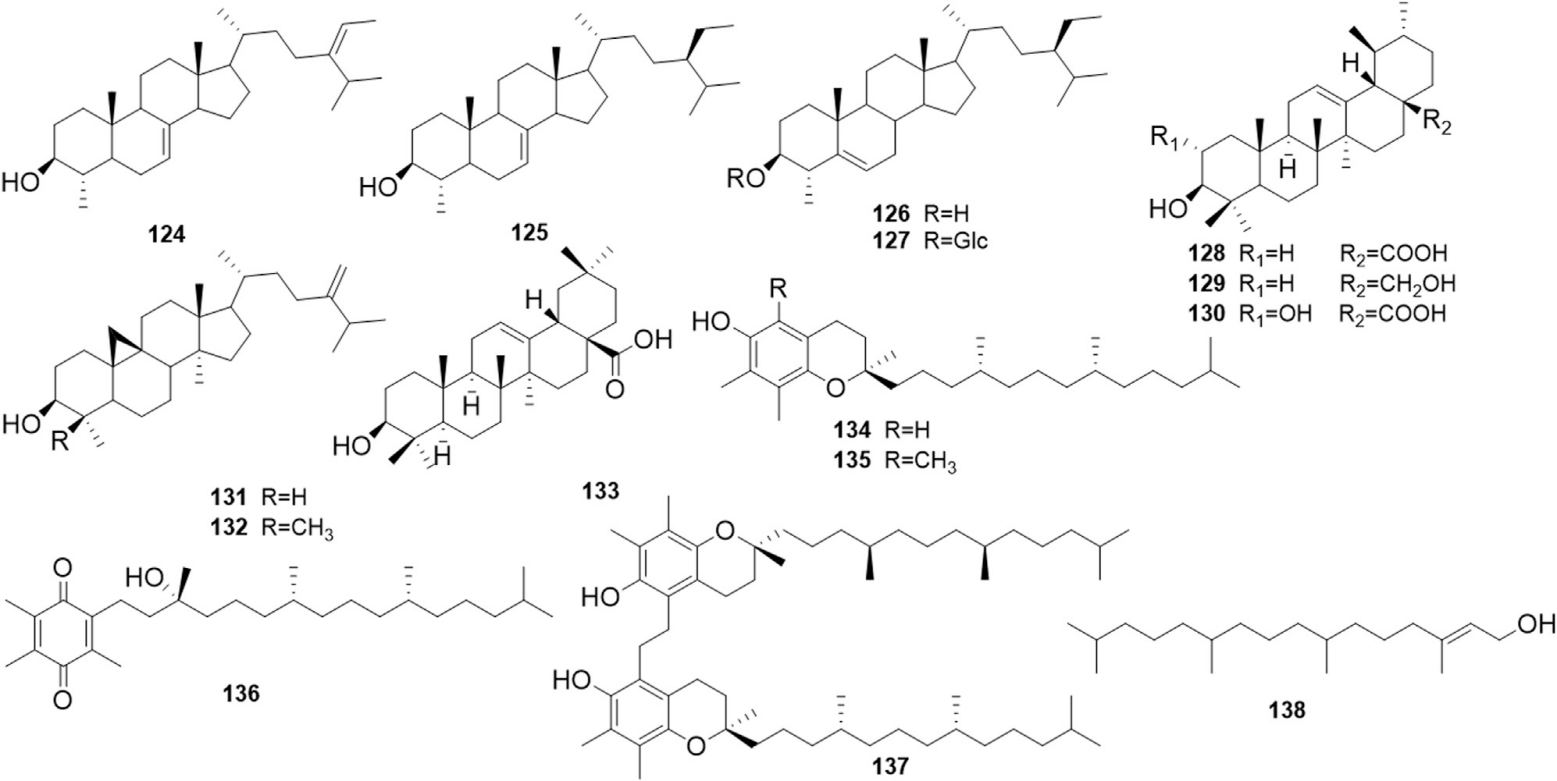
Structures of the steroids and terpenes (124–138) form *P. mume*.

**FIGURE 7:**
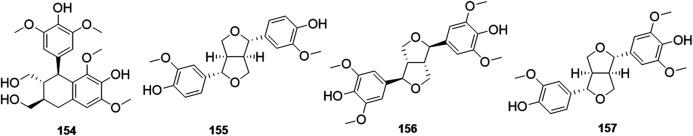
Structures of the lignans (154–157) form *P. mume*.

**FIGURE 8:**
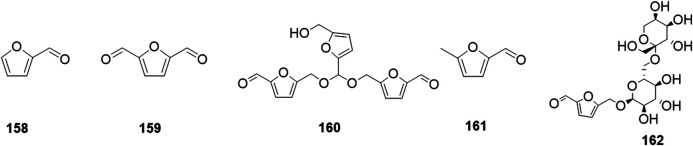
Structures of the furfurals (158–162) form *P. mume*.

**FIGURE 9:**
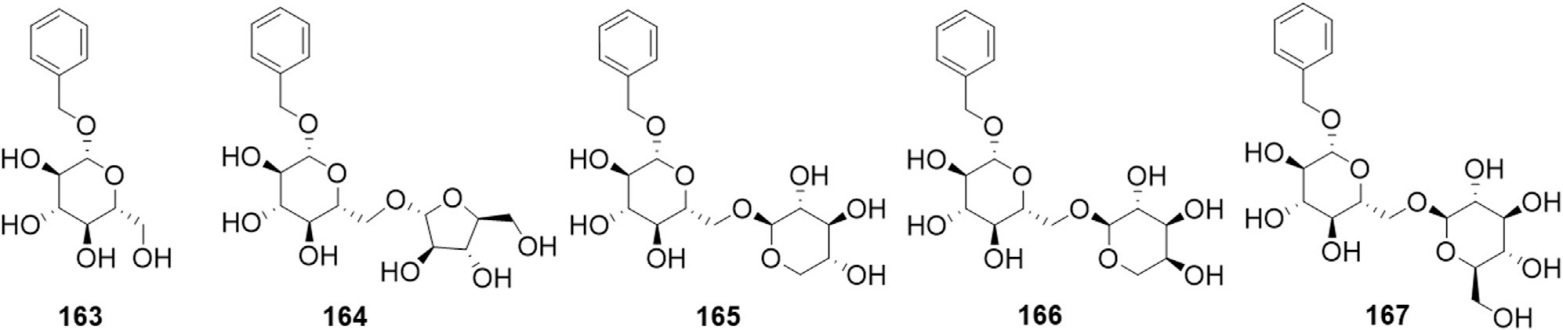
Structures of the benzyl glycosides (163–167) form *P. mume*.

**FIGURE 10:**
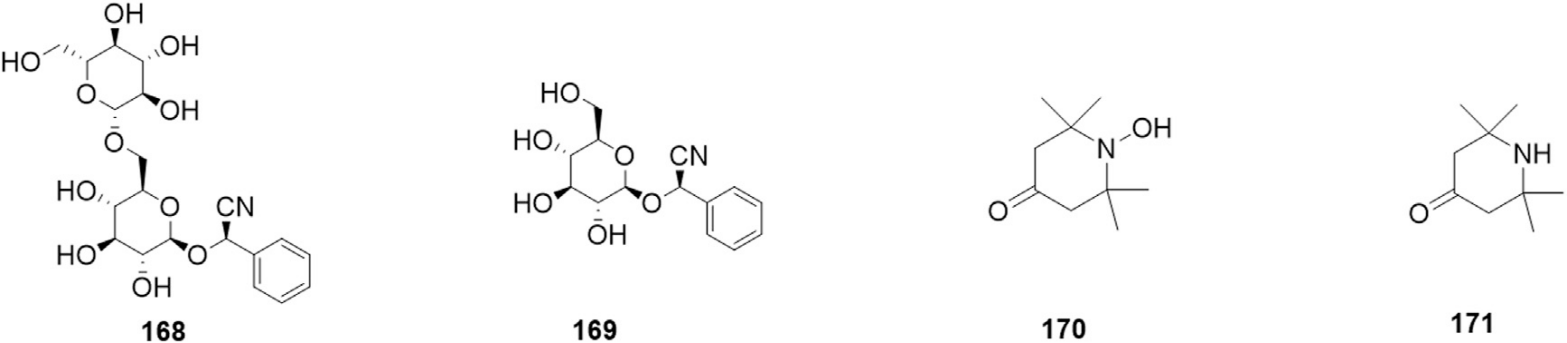
Structures of the cyanogenic glycosides (168–171) form *P. mume*.

Phytochemical studies of *P. mume* have led to the isolation and identification of many types of natural products, including phenolics, organic acids, steroids, terpenes, lignans, furfurals, benzyl glycosides, cyanogenic glycosides, and alkaloids from different parts of *P. mume*. In total, 192 chemical compounds found in the *P. mume* were listed in Table 1. The phenolic components, abundantly present in *P. mume* flower, flower bud, fruit, wood, petal, and seed, are complex, and can be subdivided into phenylpropanoid sucrose esters, hydroxycinnamoylquinic acid derivatives, flavonoids, and other phenolics; the structures of these compounds (1–96) are shown in Figures 1–4. The fruit is the most studied part of the plant, contains mainly therapeutic chemical compounds including organic acid (compounds 92–123), steroids and terpenes (compounds 124–138), lignans (compounds 154–157), furfurals (compounds 158–162), benzyl glycosides (compounds 163–167), cyanogenic glycosides and alkaloid (compounds 168–171). The structures of these compounds are shown in Figures 5–10, respectively.

**TABLE 1:** List of 192 compounds isolated from *P. mume*.

No	Compounds name	Chemical formula	Source	Content (%w/w)	Harvest region	References
*Phenylpropanoid sucrose esters*						
1	Prunose I	C_31_ H_38_ O_18_	Flowers	0.016	Japan	Yoshikawa et al. (2002)
2	Prunose II	C_29_ H_36_ O_17_	Flower	0.0084	Japan	Yoshikawa et al. (2002)
3	Prunose III	C_29_ H_36_ O_17_	Flowers	0.0084	Japan	Fujimoto et al. (2014)
4	Mumeose A	C_23_ H_30_ O_14_	Flower buds	0.00032	China	Nakamura et al. (2013a)
5	Mumeose B	C_25_ H_32_ O_15_	Flower buds	0.0010	China	Nakamura et al. (2013a)
6	Mumeose C	C_27_ H_34_ O_16_	Flower buds	0.00050	China	Nakamura et al. (2013a)
7	Mumeose D	C_31_ H_38_ O_18_	Flower buds	0.00047	China	Nakamura et al. (2013a)
8	Mumeose E	C_31_ H_38_ O_18_	Flower buds	0.00032	China	Nakamura et al. (2013a)
9	Mumeose F	C_27_ H_34_ O_16_	Flower buds	0.0011	China	Fujimoto et al. (2013)
10	Mumeose G	C_27_ H_34_ O_16_	Flower buds	0.0017	China	Fujimoto et al. (2013)
11	Mumeose H	C_27_ H_34_ O_16_	Flower buds	0.0005	China	Fujimoto et al. (2013)
12	Mumeose I	C_29_ H_36_ O_17_	Flower buds	0.0003	China	Fujimoto et al. (2013)
13	Mumeose J	C_33_ H_40_ O_19_	Flower buds	0.0033	China	Fujimoto et al. (2013)
14	Mumeose K	C_25_ H_32_ O_15_	Flower buds	0.0012	China	Nakamura et al. (2013b)
15	Mumeose L	C_29_ H_36_ O_17_	Flower buds	0.0008	China	Nakamura et al. (2013b)
16	Mumeose M	C_31_ H_38_ O_18_	Flower buds	0.0027	China	Nakamura et al. (2013b)
17	Mumeose N	C_31_ H_38_ O_18_	Flower buds	0.0043	China	Nakamura et al. (2013b)
18	Mumeose O	C_31_ H_38_ O_18_	Flower buds	0.0003	China	Nakamura et al. (2013b)
19	Mumeose *p*	C_23_ H_30_ O_14_	Flower buds	0.0002	China	Fujimoto et al. (2014)
20	Mumeose Q	C_23_ H_30_ O_14_	Flower buds	0.0003	China	Fujimoto et al. (2014)
21	Mumeose R	C_23_ H_30_ O_14_	Flower buds	0.0010	China	Fujimoto et al. (2014)
22	Mumeose S	C_23_ H_30_ O_14_	Flower buds	0.0020	China	Fujimoto et al. (2014)
23	Mumeose T	C_29_ H_36_ O_17_	Flower buds	0.0002	China	Fujimoto et al. (2014)
24	Mumeose U	C_29_ H_36_ O_17_	Flower buds	0.0012	China	Fujimoto et al. (2014)
25	Mumeose V	C_31_ H_38_ O_18_	Flower buds	0.0054	China	(Fujimoto et al., 2014)
26	2,3,4′,6′-tetra-O-acetyl-3-*O-(E)-p*-coumaroylsucrose	C_29_ H_36_ O_17_	Flower buds	0.0127	China	Fujimoto et al. (2013)
27	3-O-Feruloylsucrose	C_22_ H_30_ O_14_	Fruits	0.00004	Korea	Yan et al. (2014a)
28	*α*-d-Glucopyranoside, 3-O-[3-(4-hydroxyphenyl)-1-oxo-2-propenyl]-*β*-d-fructofuranosyl	C_21_ H_28_ O_13_	Fruits	0.00002	Korea	Yan et al. (2014b)
29	Chlorogenic acid	C_16_ H_18_ O_9_	Flowers	0.0006	Japan	Yan et al. (2014c)
30	Chlorogenic acid methyl ester	C_17_ H_20_ O_9_	Flower buds	0.11	China	Nakamura et al. (2013a)
31	5-*O-(E)-p*-coumaroylquinic acid methyl ester	C_17_ H_20_ O_8_	Flower buds	0.0014	China	Nakamura et al. (2013a)
32	5-*O-(E)-p*-coumaroylquinic acid ethyl ester	C_18_ H_22_ O_8_	Flower buds	0.0031	China	Nakamura et al. (2013a)
33	Mumeic acid-A methyl ester	C_24_ H_24_ O_10_	Flower buds	0.0034	China	Nakamura et al. (2013a)
34	Mumeic acid-A	C_23_ H_22_ O_10_	Flower buds	0.0039	China	Nakamura et al. (2013a)
35	5-O-(E)-feruloylquinic acid methyl ester	C_18_ H_22_ O_9_	Flower buds	0.0013	China	Nakamura et al. (2013a)
36	*trans*-Chlorogenic acid	C_16_ H_18_ O_9_	Flower buds	0.11	China	Nakamura et al. (2013a)
37	Chlorogenic acid ethyl ester	C_18_ H_22_ O_9_	Flower buds	0.038	China	Nakamura et al. (2013a)
38	5-*O-(E)-p*-coumaroyl quinic acid	C_16_ H_18_ O_8_	Flower buds	0.015	China	Nakamura et al. (2013a)
39	Isochlorogenic acid	C_16_ H_18_ O_9_	Fruits	0.00005	Korea	Yan et al. (2014a)
40	4-O-Caffeoylquinic acid	C_16_ H_18_ O_9_	Seeds	NM	NM	Xia et al. (2011)
41	4-O-Caffeoylquinic acid methyl ester	C_17_ H_20_ O_9_	Fruits	0.00025	Korea	Jin et al. (2012)
42	5-O-Caffeoylquinic acid	C_16_ H_18_ O_9_	Seeds	NM	NM	Xia et al. (2011)
43	5-O-Caffeoylquinic acid methyl ester	C_17_ H_20_ O_9_	Fruits	0.00025	Korea	Jin et al. (2012)
*Flavonoids*						
44	Rutin	C_27_ H_30_ O_16_	Flowers	NM	China	Yoshikawa et al. (2002)
45	Quercetin 3-O-neohesperidoside	C_27_ H_30_ O_16_	Flowers	0.0024	China	Yoshikawa et al. (2002)
46	2″-O-Acetylrutin	C_29_ H_32_ O_17_	Flowers	0.0039	China	Yoshikawa et al. (2002)
47	2″-O-Acetyl-3′-O-methylrutin	C_30_ H_34_ O_17_	Flowers	0.0008	China	Yoshikawa et al. (2002)
48	Quercetin 3-O-rhamnosyl (1→6)galactoside	C_27_ H_30_ O_16_	Flowers	0.0016	China	Yoshikawa et al. (2002)
49	Isorhamnetin 3-O-rhamnoside	C_22_ H_22_ O_11_	Flowers	0.0013	China	Yoshikawa et al. (2002)
50	Quercetin 3-O-(2″-O-acetyl)-*β*-d-glucopyranoside	C_23_ H_22_ O_13_	Flower buds	0.0016	China	Nakamura et al. (2013b)
51	Isorhamnetin 3-O-*β*-d-glucopyranoside	C_22_ H_22_ O_12_	Flower buds	0.017	China	Nakamura et al. (2013b)
52	Quercetin 3-O-(6″-O-acetyl)-*β*-d-glucopyranoside	C_23_ H_22_ O_13_	Flower buds	0.0010	China	Nakamura et al. (2013b)
53	Quercetin 3-O-(6″-O-benzoyl)-*β*-d-galactopyranoside	C_28_ H_24_ O_13_	Flower buds	0.00059	China	Nakamura et al. (2013b)
54	Isorhamnetin 3-O-*β*-d-galactopyranoside	C_22_ H_22_ O_12_	Flower buds	0.0006	China	Nakamura et al. (2013b)
55	Mumeflavonoside A	C_24_ H_24_ O_13_	Flower buds	0.0004	China	Nakamura et al. (2013b)
56	Kaempferol	C_15_ H_10_ O_6_	Fruits	0.00125	China	Guo et al. (2009)
57	Isoquercitrin	C_21_ H_20_ O_12_	Fruits	0.00007	NM	Yan et al. (2014a)
58	Quercetin	C_15_ H_10_ O_7_	Flowers	NM	China	Zhang et al. (2008)
59	Isoquercitrin	C_21_ H_20_ O_12_	Flowers	NM	China	Zhang et al. (2008)
60	Kaempferol-3-O-*β*-d-galactopyranoside	C_21_ H_20_ O_11_	Flowers	NM	China	Zhang et al. (2008)
61	Isorhamnetin	C_16_ H_12_ O_7_	Flowers	NM	China	Zhang et al. (2008)
62	Genkwanin	C_16_ H_12_ O_5_	Wood	NM	NM	Poonam et al. (2011)
63	Flavone,7-hydroxy-3,4′,5-trimethoxy-,*β*-d-glucopyranoside	C_24_ H_26_ O_11_	Wood	NM	NM	Hasegawa (1959)
64	Mumenin	C_22_ H_22_ O_11_	Wood	NM	NM	Hasegawa (1959)
65	Prudomenin	C_23_ H_24_ O_12_	Wood	NM	NM	Hasegawa (1969)
66	Genistein	C_15_ H_10_ O_5_	Fruits	0.00055	China	Guo et al. (2009)
67	2*β*,3*β*-epoxy-5,7,3′,4' -tetrahydroxyflavan-(4*α*→8)-epicatechin	C_30_ H_24_ O_12_	Fruits	0.00002	NM	Yan et al. (2014a)
68	2*β*,3*β*-epoxy-5,7,4′-trihydroxyflavan-(4*α*→8)-epicatechin	C_30_ H_24_ O_11_	Fruits	0.00004	NM	Yan et al. (2014a)
69	Cyanidin 3-rutinoside	C_27_ H_31_ O_15_	Petals	2.6	China	Zhao et al. (2006)
70	Cyanidin3-O-(6″- *O-α*-rhamnopyranosyl-*β*-glucopyranoside	C_34_ H_35_ O_20_	Petals	5.0	China	Zhao et al. (2006)
71	Cyanidin 3-O-(6″ -O-galloyl-*β*-glucopyranoside)	C_28_ H_25_ O_15_	Petals	NM	China	Zhao et al. (2004)
72	Cyanidin3-O-(6″ -O-E-feruloyl-*β*-glucopyranoside)	C_31_ H_29_ O_14_	Petals	NM	China	Zhao et al. (2004)
73	(-)-Epicatechin	C_15_ H_14_ O_6_	Fruits	0.00022	NM	Yan et al. (2014a)
74	Leucocyanidol	C_15_ H_14_ O_7_	Fruits	NM	NM	Poonam et al. (2011)
75	(+)-cyanidanol	C_15_ H_14_ O_6_	Wood	NM	NM	Hasegawa (1959)
76	Naringenine	C_15_ H_12_ O_5_	Wood	NM	NM	Hasegawa (1959)
77	Liquiritigenin-7-O-*β*-D-glucoside	C_21_ H_22_ O_9_	Fruits	0.00015	Korea	Jin et al. (2012)
78	Prunin	C_21_ H_22_ O_10_	Wood	NM	NM	Hasegawa (1959)
79	Flavanone,3,5,7-trihydroxy-4′,8-dimethoxy-,7-*β*-d-glucopyranoside	C_23_ H_26_ O_12_	Wood	NM	NM	Hasegawa (1969)
*Other phenolics*						
80	(E)-caffeic acid	C_9_ H_8_ O_4_	Flower buds	0.0016	China	Fujimoto et al. (2013)
81	*(E)-p*-coumaric acid	C_9_ H_8_ O_3_	Flower buds	0.0007	China	Fujimoto et al. (2013)
82	(E)-ferulic acid	C_10_ H_10_ O_4_	Flower buds	0.0015	China	Fujimoto et al. (2013)
83	(S,R)-1-O-Caffeoylglycerol	C_12_ H_14_ O_6_	Flower buds	0.0011	China	Fujimoto et al. (2013)
84	(S,R)-1-O-Feruloylglycerol	C_13_ H_16_ O_6_	Flower buds	0.0043	China	Fujimoto et al. (2013)
85	Methyl (E)-4-hydroxycinnamate	C_10_ H_10_ O_3_	Fruits	0.00003	Korea	Yan et al. (2014a)
86	*o*-Cresol	C_7_ H_8_ O	Fruits	NM	NM	Kameoka and Kitagawa (1976)
87	*p*-Cresol	C_7_ H_8_ O	Fruits	NM	NM	Kameoka and Kitagawa (1976)
88	Guaiacol	C_7_ H_8_ O_2_	Fruits	NM	NM	Kameoka and Kitagawa (1976)
89	Protocatechoic acid	C_7_ H_6_ O_4_	Fruits	NM	NM	Cho et al. (2019)
90	Syringic acid	C_9_ H_10_ O_5_	Fruits	NM	NM	Cho et al. (2019)
91	*p*-Tyrosol	C_8_ H_10_ O_2_	Fruits	0.00005	Korea	Yan et al. (2014a)
92	1-O-(3-hydroxy-4-methoxybenzoyl)-*β*-d-glucopyranose	C_14_ H_18_ O_9_	Flower buds	0.0005	China	Fujimoto et al. (2013)
93	Eugenol	C_10_ H_12_ O_2_	Fruits	NM	Japan	Miyazawa et al. (2009)
94	Prunate	C_19_ H_22_ O_6_	Fruits	0.00001	Korea	Jeong et al. (2006)
95	*cis-p*-coumaric acid	C_9_ H_8_ O_3_	Fruits	NM	Japan	Mitani et al. (2013)
96	Hypericin	C_30_ H_16_ O_8_	Flowers	NM	China	Zhang et al. (2008)
*Organic acids*						
97	2-Monomethyl citrate	C_7_ H_10_ O_7_	Fruits	0.00420	Korea	Yan et al. (2014b)
98	1,5-Dimethyl citrate	C_8_ H_12_ O_7_	Fruits	0.00042	Korea	Yan et al. (2014c))
99	Benzoic acid	C_7_ H_6_ O_2_	Flowers	NM	China	Zhang et al. (2008)
100	Propanedioic acid	C_3_ H_4_ O_4_	Fruits	NM	China	Guo et al. (2007)
101	Citric acid	C_6_ H_8_ O_7_	Fruits	0.00073	China	Wang et al. (2019)
102	Succinic acid	C_4_ H_6_ O_4_	Fruits	NM	China	Gao (2012)
103	Maleic acid	C_4_ H_4_ O_4_	Fruits	NM	China	Gao (2012)
104	Fumaric acid	C_4_ H_4_ O_4_	Fruits	NM	China	Gao (2012)
105	Oxalic acid	C_2_ H_2_ O_4_	Fruits	NM	China	Gao (2012)
106	Ascorbic acid	C_6_ H_8_ O_6_	Fruits	NM	China	Gao (2012)
107	Acetic acid	C_2_ H_4_ O_2_	Fruits	NM	China	Gao (2012)
108	Malic acid	C_4_ H_6_ O_5_	Fruits	NM	China	Gao (2012)
109	Tartaric acid	C_4_ H_6_ O_6_	Fruits	NM	China	Gao (2012)
110	Lactic acid	C_3_ H_6_ O_3_	Unknown			Chen et al. (2006)
111	Lauric acid	C_12_ H_24_ O_2_	Fruits	NM	NM	Kameoka and Kitagawa (1976)
112	Valeric acid	C_5_ H_10_ O_2_	Fruits	NM	NM	Kameoka and Kitagawa (1976)
113	Caproic acid	C_6_ H_12_ O_2_	Fruits	NM	NM	Kameoka and Kitagawa (1976)
114	Stearic acid	C_18_ H_36_ O_2_	Fruits	NM	NM	Kameoka and Kitagawa (1976)
115	Butyric acid	C_4_ H_8_ O_2_	Fruits	NM	China	Guo et al. (2007)
116	Heptanoic acid	C_7_ H_14_ O_2_	Fruits	NM	China	Guo et al. (2007)
117	2-Methylbutanoic acid	C_5_ H_10_ O_2_	Fruits	NM	China	Guo et al. (2007)
118	Palmitic acid	C_16_ H_32_ O_2_	Fruits	NM	Japan	Miyazawa et al. (2009)
119	Linolenic acid	C_18_ H_30_ O_2_	Fruits	NM	China	Guo et al. (2007)
120	3-Methylbutanoic acid	C_5_ H_10_ O_2_	Fruits	NM	China	Guo et al. (2007)
121	Linoleic acid	C_18_ H_32_ O_2_	Fruits	NM	China	Guo et al. (2007)
122	Formic acid	C H_2_ O_2_	Fruits	NM	China	Guo et al. (2007)
123	Propionic acid	C_3_ H_6_ O_2_	Fruits	NM	China	Guo et al. (2007)
*Steroids and terpenes*						
124	Citrostadienol	C_30_ H_50_ O	Fruits	0.00002	Korea	Yan et al. (2015)
125	24-Ethyl-lophenol	C_30_ H_52_ O	Fruits	0.00004	Korea	Yan et al. (2015)
126	*β*-sitosterol	C_29_ H_50_ O	Fruits	0.0014	Korea	Yan et al. (2015)
127	Daucosterol	C_35_ H_60_ O_6_	Fruits	0.00305	Korea	Yan et al. (2015)
128	Ursolic acid	C_30_ H_48_ O_3_	Fruits	0.00455	Korea	Yan et al. (2015)
129	Uvaol	C_30_ H_50_ O_2_	Fruits	0.00013	Korea	Yan et al. (2015)
130	Corosolic acid	C_30_ H_48_ O_4_	Fruits	0.00015	Korea	Yan et al. (2015)
131	Cycloeucalenol	C_30_ H_50_ O	Fruits	0.00005	Korea	Yan et al. (2015)
132	24-Methylenecycloartanol	C_31_ H_52_ O	Fruits	0.00038	Korea	Yan et al. (2015)
133	Oleanolic acid	C_30_ H_48_ O	Fruits	NM	NM	Poonam et al. (2011)
134	*γ*-tocopherol	C_28_ H_48_ O_2_	Fruits	0.00012	Korea	Yan et al. (2015)
135	*α*-tocopherol	C_29_ H_50_ O_2_	Fruits	0.00044	Korea	Yan et al. (2015)
136	*α*-tocopherylquinone	C_29_ H_50_ O_3_	Fruits	0.00002	Korea	Yan et al. (2015)
137	1,2-Bis(*γ*-tocopherol-5-yl)ethane	C_58_ H_98_ O_4_	Fruits	0.00003	Korea	Yan et al. (2015)
138	Phytol	C_20_ H_40_ O	Flowers	NM	NM	Poonam et al. (2011)
*Amino acids*						
139	l-aspartic acid	C_4_ H_7_ N O_4_	Fruits	0.0000005	Korea	Kim et al. (2014)
140	*α*-amino-*n*-butyric acid	C_4_ H_9_ N O_2_	Fruits	0.0000006	Korea	Kim et al. (2014)
141	*Glycine*	C_2_ H_5_ N O_2_	Fruits	0.00000004	Korea	Kim et al. (2014)
142	l-alannine	C_3_ H_7_ N O_2_	Fruits	0.0000004	Korea	Kim et al. (2014)
143	l-Serine	C_3_ H_7_ N O_3_	Fruits	0.000001	Korea	Kim et al. (2014)
144	l-glutamic acid	C_5_ H_9_ N O_4_	Fruits	0.0000004	Korea	Kim et al. (2014)
145	l-Lysine	C_6_ H_14_ N_2_ O_2_	Fruits	0.00000008	Korea	Kim et al. (2014)
146	l-Leucine	C_6_ H_13_ N O_2_	Fruits	0.0000002	Korea	Kim et al. (2014)
147	l-Phenylalanine	C_9_ H_11_ N O_2_	Fruits	0.00000002	Korea	Kim et al. (2014)
148	l-Asparagine	C_4_ H_8_ N_2_ O_3_	Fruits	0.00001	Korea	Kim et al. (2014)
149	l-Histidine	C_6_ H_9_ N_3_ O_2_	Fruits	0.00000015	Korea	Kim et al. (2014)
150	l-Valine	C_5_ H_11_ N O_2_	Fruits	0.0000002	Korea	Kim et al. (2014)
151	l-Threonine	C_4_ H_9_ N O_3_	Fruits	0.0000003	Korea	Kim et al. (2014)
152	l-isoleucine	C_6_ H_13_ N O_2_	Fruits	0.00000002	Korea	Kim et al. (2014)
153	l-Arginine	C_6_ H_14_ N_4_ O_2_	Fruits	0.00000029	Korea	Kim et al. (2014)
*Lignans*						
154	(+)-lyoniresinol	C_22_ H_28_ O_8_	Fruits	0.00013	Korea	Yan et al. (2014a)
155	(+)-pinoresinol	C_20_ H_22_ O_6_	Fruits	0.00002	Korea	Yan et al. (2014a)
156	(+)-syringaresinol	C_22_ H_26_ O_8_	Fruits	0.00022	Korea	Yan et al. (2014a)
157	(+)-mediaresinol	C_21_ H_24_ O_7_	Fruits	0.00003	Korea	Yan et al. (2014a)
*Furfurals*						
158	Furfural	C_5_ H_4_ O_2_	Fruits	NM	Japan	Miyazawa et al. (2009)
159	5-Hydroxymethyl-2-furaldehyde	C_6_ H_6_ O_3_	Fruits	0.001	Korea	Jin et al. (2012)
160	5-Hydroxymethyl-2-furaldehydebis (5-formylfurfuryl) acetal	C_18_ H_16_ O_8_	Fruits	NM	Korea	Jang et al. (2018)
161	5-Methyl-2-furfural	C_6_ H_6_ O_2_	Fruits	NM	NM	Kameoka and Kitagawa (1976)
162	5-[*β*-d-Fructopyranosyl-(2→6)-*α*-D-glucopyranosyloxymethyl]-2-furancarboxaldehyde	C_18_ H_26_ O_13_	Fruits	0.00006	Korea	Yan et al. (2014a)
*Benzyl glycosides*						
163	Benzyl-*β*-d-glucopyranoside	C_13_ H_18_ O_6_	Flowers	0.0022	Korea	Yan et al. (2014a)
164	Phenylmethyl6-*O-α*-l-arabinofuranosyl-*β*-d-glucopyranoside	C_18_ H_26_ O_10_	Fruits	0.00883	Korea	Yan et al. (2014a)
165	Benzyl *β*-primeveroside	C_18_ H_26_ O_10_	Fruits	0.00242	Korea	Yan et al. (2014a)
166	Benzylalcohol *O-α*-l-arabinopyranosyl-(1→6)-*β*-d-glycopyranoside	C_18_ H_26_ O_10_	Fruits	0.00012	Korea	Yan et al. (2014a)
167	Benzyl gentiobioside	C_19_ H_28_ O_11_	Fruits	0.00012	Korea	Yan et al. (2014a)
*Cyanogenic glycosides*						
168	Amygdalin	C_20_ H_27_ N O_11_	Fruits	0.00116	China	Wang et al. (2019)
169	Prunasin	C_14_ H_17_ N O_6_	Fruits	0.0002	Korea	Jin et al. (2012)
*Alkaloids*						
170	2,2,6,6-Tetramethyl-4-oxo-1-piperidinooxy	C_9_ H_16_ N O_2_	Fruits	NM	NM	Ren et al. (2004)
171	Triacetonamine	C_9_ H_17_ N O	Fruits	NM	NM	Ren et al. (2004)
*Other compounds*						
172	Phytol	C_20_ H_40_ O	Flowers	0.0009	Japan	Yoshikawa et al. (2002)
173	Eugenyl glucoside	C_16_ H_22_ O_7_	Flowers	0.050	Japan	Yoshikawa et al. (2002)
174	Chavicol *β*-D-glucoside	C_15_ H_20_ O_6_	Flowers	0.0014	Japan	Yoshikawa et al. (2002)
175	*β*-d-Glucopyranosyl benzoate	C_13_ H_16_ O_7_	Fruits	0.00015	Korea	Yan et al. (2014a)
176	3,4,5-Trimethoxyphenyl-*β*-d-glucopyranoside	C_15_ H_22_ O_9_	Fruits	0.00039	Korea	Yan et al. (2014a)
177	Rhodioloside E	C_21_ H_38_ O_11_	Fruits	0.00008	Korea	Yan et al. (2014a)
178	Benzaldehyde	C_7_ H_6_ O	Fruits	NM	Japan	Miyazawa et al. (2009)
179	Linalool	C_10_ H_18_ O	Fruits	NM	Japan	Miyazawa et al. (2009)
180	*α*-terpineol	C_10_ H_18_ O	Fruits	NM	Japan	Miyazawa et al. (2009)
181	*p*-Cymene	C_10_ H_14_	Fruits	NM	Japan	Miyazawa et al. (2009)
182	Squalene	C_30_ H_50_	Fruits	0.00050	China	Wang et al. (2019)
183	Ceryl alcohol	C_26_ H_54_ O	Fruits	NM	NM	Poonam et al. (2011)
184	Benzyl alcohol	C_7_ H_8_ O	Fruits	NM	NM	Kameoka and Kitagawa (1976)
185	Isoamyl alcohol	C_5_ H_12_ O	Fruits	NM	NM	Kameoka and Kitagawa (1976)
186	2,3-Dimethylmaleic anhydride	C_6_ H_6_ O_3_	Fruits	NM	NM	Kameoka and Kitagawa (1976)
187	*cis*-3-hexen-1-ol	C_6_ H_12_ O	Fruits	NM	NM	Kameoka and Kitagawa (1976)
188	Ethyl benzoate	C_9_ H_10_ O_2_	Fruits	NM	NM	Kameoka and Kitagawa (1976)
189	Patchouli alcohol	C_15_ H_26_ O	Fruits	NM	NM	Ichikawa et al. (1989)
190	Rhodioloside E	C_21_ H_38_ O_11_	Fruits	0.00008	Korea	Yan et al. (2014a)
191	*α*-methoxy-2,5-furandimethanol	C_7_ H_10_ O_4_	Fruits	0.00005	Korea	Yan et al. (2014b)
192	Butyl glucoside	C_10_ H_20_ O_6_	Fruits	0.0011	Korea	Yan et al. (2014c)

NM = Not mentioned in ref.

### Pharmacological Properties and Clinical Applications

#### Metabolic Diseases

##### Diabetes Mellitus

DM is one of the most common metabolic diseases in the world. It is characterized by hyperglycemia as result of abnormal insulin secretion and insulin resistance. Obesity, which is normally associated with hyperglycemia and insulin resistance, is a high-risk factor for DM. One study found that a water extract of *P. mume* fruit and *Lithospermum erythrorhizon* root synergistically improved insulin sensitivity and prevented visceral adiposity in a high-fat diet (HFD)-fed ovariectomized rats model (Ko et al., 2013). A 70% ethanol extract of *P. mume* fruit was described as able to increas glucose uptake in C2C12 myotubes by activating the peroxisome proliferator-activated receptor (PPAR)-γ. The extract also significantly improved fasting glucose levels and glucose intolerance, reduced body weight, liver and adipose tissue weight without affecting food intake in HFD-fed mice (Hwang et al., 2012; Shin et al., 2013). A 70% ethanol extract of *P. mume* leaves also decreased blood glucose levels in a dose-dependent manner, and the polyphenol compounds were conjectured to account for these activities (Lee et al., 2016). In another study, phenolic extracts of *P. mume* inhibited small intestinal disaccharidase activity and suppressed postprandial elevation of blood glucose levels in rats (Kishida et al., 2013). A TCM formula called “wumei wan” in Chinese has also been reported to improve insulin resistance (IR) in type 2 DM rats, which might be related to up-regulation of protein and mRNA expression levels of the insulin receptor (Insr), insulin receptor substrate 1 (Irs-1), glucose transporter4 (Glut-4), and *β*-arrestin-2 in the liver and skeletal muscle (Li et al., 2013).

Clinically, in a multicenter randomized controlled pilot trial, 85 subjects diagnosed with type 2 DM, were randomized to receive either *wumei wan* or metformin. After a12-weeks intervention, the *P. mume* formula *wumei wan* decreased the fasting plasma glucose (FPG), postprandial glucose (PPG), and glycosylated hemoglobin (HbAlc) levels as effectively as the hypoglycemic agent metformin. In addition, the formula could significantly decrease the body mass index (BMI) when the patient’s BMI was greater than 23, but not when the BMI was below 23 (Tu et al., 2013).

Furthermore, the phenolic compounds 9∼13, 29, 30, 33, 34, 47 extracts from the flower buds of *P. mume* inhibited aldose reductase, reduced sorbitol accumulation in eye lenses and retinas, and had the potential to prevent diabetic complications such as cataract (Yoshikawa et al., 2002; Fujimoto et al., 2013).

Altogether, these studies demonstrated that *P. mume* could prevent obesity, maintain glucose metabolism, prevent diabetic complications, and bring therapeutic benefit to the patients with type 2 DMs.

##### Hypolipidemic Effects

Squalene synthase plays an important role in the cholesterol biosynthesis pathway. Inhibiting this enzyme in hypercholesterolemia can lower not only plasma cholesterol but also plasma triglyceride levels. Chlorogenic acid isolated from *P. mume* fruit inhibited squalene synthase in pig liver homogenate with an IC_50_ level of 100 nm (Choi et al., 2007). In HFD-fed mice, the 70% ethanol extract of *P. mume* fruit decreased serum triglyceride (TG) levels significantly (Hwang et al., 2012; Shin et al., 2013). Furthermore, in HFD-fed rats fed with *P. mume* concentrate for four°weeks, the total lipid, total cholesterol, and TG serum levels, and the atherogenic index decreased significantly compared with the HFD model control group; while, the serum level of high-density lipoprotein (HDL)-cholesterol was significantly higher than the HFD model control group (Chyun et al., 2012). Therefore, the plant may act as a potential therapeutic agent for hypercholesterolemia.

##### Gout

Gout is a metabolic disorder characterized by recurrent acute arthritis, hyperuricemia, and deposition of sodium urate in and around the joints, sometimes with the formation of uric acid calculi. The enzyme xanthine oxidase (XO) can oxidize hypoxanthine and xanthine to uric acid, thus playing an important role in the catabolism of purines, which are associated with the metabolic disorders of hyperuricemia and gout (Wang et al., 2010). *P. mume* can be used to treat gouty arthritis in combination with other herbal medicines based on clinical experience in TCM (Chen et al., 2007). Animal studies have also shown that a methanol extract of *P. mume* fruit, with the seeds removed (70 and 140 mg/kg, 7°days) decreased serum and liver uric acid levels, elevated urinary uric acid levels, and reduced hepatic XO activity in mice with potassium oxonate induced hyperuremia (Yi et al., 2012).

##### Osteoporosis

Osteoporosis is a metabolic disease that frequently occurs in aging communities. This degenerative disease is characterized by a progressive loss of bone mineral density (BMD) and deterioration of the bone micro-architecture, causing an increased risk of fracture (Bi et al., 2006). Currently, therapies for osteoporosis are focused on inhibiting osteoclastic activity, stimulating osteoblastic activity, and decreasing oxidative stress (Arai et al., 2007). MC3T3-E1 is a classic cell model of the osteoblastic phenotype. Treatment of MC3T3-E1 cells with the water-soluble fraction of *P. mume* increased osteogenic mRNA expression of bone morphogenetic protein (BMP-2), osteopontin (OPN), RUNX2, and increased alkaline phosphatase (ALP) activity, which is a marker of the early period of osteoblastic differentiation, and therefore induced cell proliferation and differentiation. Moreover, the Alizarin Red staining assay demonstrated that *P. mume* increased calcium deposition, and therefore had an accelerative effect on the mineralization of cells (Kono et al., 2011). Other research groups have explored the antioxidant and anti-osteoporosis activities of compounds isolated from *P. mume* fruit using murine pre-osteoblastic MC3T3-E1 cells and pre-osteoclastic RAW 264.7cells. These studies showed that phenolic and lignans compounds such as compounds 19∼21, 30, 39, 154∼156 exhibited peroxyl radical-scavenging activities in a dose-dependent manner. The benzyl glycoside compound 166 and flavonoid compounds 44, 45, 57, 67, 68, 73 significantly stimulated the differentiation of pre-osteoblastic MC3T3-E1 cells by increasing collagen synthesis and mineralization (Yan et al., 2014a; 2014b). Moreover, some phenylpropanoid sucrose esters, organic acids, lignans and glycoside compounds 30, 39, 98, 154, 163, 169 possessed significant inhibitory activity against osteoclast differentiation by suppressing tartrate-resistant acid phosphatase (TRAP) activity in pre-osteoclastic RAW 264.7cells (Yan et al., 2015). These results show that *P. mume* may be an excellent source of anti-osteoporosis activity that can be used to prevent osteoporosis.

### Digestive System Diseases

#### Liver Protection

In recent years, many preclinical studies have demonstrated the antioxidant (Xia et al., 2010; Kang et al., 2016) and anti-inflammatory (Morimoto et al., 2009; Mitani et al., 2013) effects of *P. mume*. Oxidative stress and inflammatory reactions are key risk factors of some chronic liver diseases, such as alcoholic liver disease, non-alcoholic fatty liver disease (NAFLD), and viral hepatitis (Oyanagi et al., 1999; Lieber, 2001; Ekstedt et al., 2006), thus the hepatoprotective effects of *P. mume* have been investigated in both animal and clinical models.

MK615 is a commercial product extract from the fruit of *P. mume* that is rich in hydrophobic substances. Hokari (2012) revealed that d-galactosamine hydrochloride (D-GalN) (600 mg/kg, single intraperitoneal injection) induced hepatopathy in a rat model. MK615 treatment (4 ml/kg per day for 7°days) significantly decreased alanine aminotransferase (ALT) and aspartate aminotransferase (AST) plasma levels and reduced hepatic injury. In the same report, a case series study was carried out to evaluate the clinical effects of MK615. Fifty-eight enrolled patients with liver disorders, including hepatitis C, NAFLD, and autoimmune liver disease, orally took MK615 solution 13 g every day for 12°weeks. After a 12-weeks intervention, the serum ALT and AST levels of these patients decreased significantly compared with the pretreatment baseline levels.

In another randomized double-blind placebo-controlled study, 45 healthy subjects with transaminase levels between 20 and 40°UI/L were enrolled, and two doses of a food supplement containing a standardized extract of *P. mume* were administered. After 3°months of treatment, the liver enzyme (ALT, AST, and gamma-glutamyl transferase [γGT]) levels, lipid profile parameters (HDL cholesterol, LDL/HDL ratio, and triglycerides), glycemia, oxidative parameters (reduced or oxidized plasma cysteine (Cys), plasma CysGly, erythrocyte glutathione (GSH), plasma GSH, and plasma neopterin/creatinine ratio) were significantly improved vs. the placebo group and the pretreatment baseline (Beretta et al., 2016).

In the alcoholic liver injury mouse model, the *P. mume* formula also exhibited hepatoprotective effects (Chen et al., 2016). Khan et al. (2017) investigated the molecular mechanism using a metabolic approach. The three-way hierarchical cluster analysis showed that 101 features were statistically different among the alcohol and *P. mume* pretreatment groups. The relative concentrations of compounds such as phosphatidylcholine and Saikosaponin BK1 increased significantly in the *P. mume* treatment group. These compounds are responsible for the hepatoprotective effects of *P. mume* by inhibiting the reactive oxygen species (ROS)-mediated p53 and mitogen-active protein kinase (MAPK) signaling pathways.

#### *Helicobacter pylori*-Related Chronic Gastritis

Epidemiological evidence has indicated a significant relationship between *Helicobacter pylori* (*H. pylori*) infection and chronic gastritis (Uemura et al., 2001). Some studies have found that *P. mume* extract has direct bactericidal activity against *H. pylori* both *in vitro* and *in vivo* (Fujita et al., 2002; Otsuka et al., 2005; Miyazawa et al., 2006). A clinical case series study, which enrolled 18 *H. pylori*-positive subjects, demonstrated that drinking 130 ml 1% concentrated fruit juice of *P. mume* twice a day for two°weeks, resulted in a slight fall in the urea breath test (UBT) values (Nakajima et al., 2006). Enomoto et al. (2010) carried out a study to examine the associations between *P. mume* intake and *H. pylori*-related chronic gastritis. The results showed that in the 458 non-elderly *H. pylori*-positive subjects (age range 30–64°years), the *H. pylori* antibody titers and serum PG-II levels were significantly lower in the high dose *P. mume* intake group compared with the low dose intake group. Thus, *P. mume* extract was shown to have a potential protective effect against *H. pylori* related chronic gastritis.

#### Dysmotility Disease

*P. mume* is believed to improve gastrointestinal dysmotility and dyspepsia in traditional medicine in Eastern countries. Some scientific studies have provided evidence for the efficacy of such folk remedies. Tamura et al. (2011) found that *P. mume* contains both soluble and insoluble fibers and can increase fecal output and fecal lipid excretion significantly. A methanol extract of *P. mume* was reported to modulate the pacemaker activities of interstitial cells of Cajal (ICCs) and was proposed as a potential gastroprokinetic agent for regulating gastrointestinal motility (Lee et al., 2017). The improvement in gastrointestinal motility also brought benefits to constipation and gastroesophageal reflux diseases (GERD), according to several animal and clinical experiments (Na et al., 2012). Jung et al. (2014) carried out a double-blind, randomized, placebo-controlled trial, in which patients experiencing constipation consumed *P. mume* fruit extract 7.2 g (*n* = 28) or a placebo (*n* = 29) twice a day for eight°weeks. The colon transit time and defecation function were evaluated by questionnaire. The results showed a significant decrease in total colon transit time and abdominal pain during defecation in the group that consumed *P. mume* compared with the placebo group. In a community cohort study, the frequency scale for symptom of GERD (FSSG) questionnaire was used to investigate the effects of *P. mume* consumption on GERD symptoms. Of a total of 1303 subjects, 392 were categorized into the *P. mume* daily intake group, 911 were included in the no or occasional intake group. The results showed that the total FSSG score and FSSG dysmotility score were significantly lower in the *P. mume* daily intake group compared with no or occasional intake (Maekita, 2015).

#### Inflammatory Bowel Disease

Oxidative stress and inflammatory reactions are the major etiologies of IBD. Oxidative stress due to excessive ROS triggers inflammatory reactions of the gut wall and causes tissue-disruptive disease (Ko and Auyeung, 2014). Many studies have reported the free radical-scavenging (Matsuda et al., 2003; Xia et al., 2010), antioxidant (Karakaya et al., 2001; Kang et al., 2016), and anti-inflammatory properties of *P. mume* (Choi et al., 2007; Morimoto et al., 2009). Some studies have assessed further beneficial effects of *P. mume* extract or formulation on different IBD mouse models. The results showed that *P. mume* treatment decreased immunoglobulin M (IgM) and immunoglobulin E (IgE) levels, reduced COX-2, tumor necrosis factor alpha (TNF-α), interferon (IFN-γ), interleukin (IL)-12, and IL-17 levels in the colon tissue of colitis mouse models, alleviated dextran sulfate sodium (DSS) or 2,4,6-trinitrobenzene sulfonic acid (TNBS)-induced histological changes and inflammatory responses (Liu et al., 2009; Zhang et al., 2011; Lee et al., 2014; Lee S. Y. et al., 2017; Kim et al., 2021). All these studies show that *P. mume* may represent a potential new therapeutic agent for IBD treatment.

### Nervous System Diseases

The human hippocampus is associated with cognitive function such as learning, memory, and emotional control (Burgess et al., 2002). A mixture of *P. mume* concentrate, disodium succinate and Span80 (3.6:4.6:1 ratios) improved the spatial memory of normal rats in the Morris water maze test, the effects being linked to the MAPK/ERK (extracellular signal-regulated kinase) signaling pathway that results in the phosphorylation of cyclic adenosine monophosphate (cAMP)-response-element-binding protein (CREB) through tropomyosin receptor kinase B (TrkB) and/or the NR2B subunit of the *N*-methyl-d-aspartate (NMDA) receptor (Kim et al., 2008).

Chronic cerebral hypoperfusion (CCH) can cause white matter and hippocampal damage and is a key etiological factor in vascular dementia (VaD). The permanent bilateral common carotid artery occlusion (BCCAo) animal model has been widely used to study CCH-relevant nervous system diseases (Farkas et al., 2007). Jeon et al. (2012) used the BCCAo rat model to study the effects of *P. mume* extract on cognitive deficits caused by CCH. The results showed that an aqueous extract of *P. mume* (200 mg/kg, 40°days) reduced microglial activation, decreased *p*-ERK expression, prevented nuclear factor kappa-light-chain-enhancer of activated B cells (NF-κB) activation in rat hippocampus, and improved the spatial learning of rats in the Morris water maze task. Likewise, an ethanol extract of *P. mume* alleviated inflammatory responses and cholinergic dysfunction by attenuating white matter lesions, decreasing expression of pro-inflammatory mediators, inhibiting microglial and astrocytic activation, and down-regulating toll-like receptor 4 (TLR4) and p38MAPK signaling (Jeon, 2015; Lee et al., 2015; Kim et al., 2016).

In addition, *P. mume* can also benefit neurodegenerative diseases such as Alzheimer’s disease. Kim et al. (2015) found that an ethanol extract of *P. mume* attenuated memory impairment in the scopolamine-induced mouse model. Park et al. (2016) examined the effects of *P. mume* on cognitive impairments in 5XFAD transgenic mice with five typical Alzheimer mutations. After a 90-days treatment, the *P. mume* group performed better in the Morris water maze task, the object/location novelty recognition test, and contextual fear-conditioning compared with the model group.

### Cardiovascular Disease

Different study groups have found that the fruitjuice concentrate of *P. mume* markedly improved blood fluidity in different micro-channel instruments (Chuda et al., 1999; Kubo et al., 2005). The polyacylated sucrose, citric acid, and mumefural derivatives of *P. mume* showed inhibitory effects on collagen-, arachidonic acid-, and ADP-induced platelet aggregation *in vitro* (Yoshikawa et al., 2002; Kubo et al., 2005). An herbal mixture of *Phyllostachys pubescens* leaves and *P. mume* fruits also showed inhibitory effects on platelet aggregation *in vitro* (Dong-Seon et al., 2013; Son et al., 2017). In the arteriovenous shunt thrombosis rat model and the carrageenan-induced mice tail thrombosis model, the mixture dose-dependently reduced the weight or length of tail thrombosis, respectively. The mechanism study showed that the mixture upregulated intracellular cAMP levels, inhibited the release of granule contents containing serotonin, platelet-activating factor (PAF), and thromboxane A2 (TXA_2_), and decreased the intracellular concentration of calcium ion. The mixture also exerted inhibitory effects by deactivating the collagen receptor glycoprotein VI (GPVI), blocking ligand binding to the receptor, inhibiting the downstream signaling pathway and the ERK activation pathway, and inhibiting the conversion of fibrinogen to fibrin (Dong-Seon et al., 2013; Son et al., 2017).

Utsunomiya et al. (2002) found that a fruit juice concentrate of *P. mume* markedly inhibited angiotensin II and H_2_O_2_-induced epidermal growth factor (EGF) receptor transactivation, inhibited ERK activation, and mitigated angiotensin II-induced vascular remodeling. The chlorogenic acid derived from *P. mume* decreased angiotensin converting enzyme (ACE) levels in rat plasma (Ina et al., 2003). Jo et al. (2019) studied the vasodilatory effects of a 70% ethanol extract of *P. mume* branches on isolated rat aortic rings. The authors showed the vasorelaxant effect of the extract was endothelium dependent. The extract affected the nitric oxide (NO) cyclic guanosine monophosphate (cGMP) pathway, the prostacyclin pathway, the muscarinic receptor pathway, potassium channels, and might represent a promising anti-hypertensive treatment.

Clinically, a 12-weeks double-blind randomized placebo-controlled pilot trial evaluated the anti-hypertensive effects of *P. mume*. The study recruited 15 participants with normal or normal high blood pressure (BP) (systolic blood pressure [SBP],130–139 mmHg; diastolic blood pressure [DBP], 85–89 mmHg) or hypertension grade 1 (SBP, 140–159 mmHg; DBP, 90–99 mmHg) and taking no anti-hypertensive agents. After a 12°weeks-intervention, the *P. mume* group showed a lower, albeit not significant, SBP compared with the control group (Takemura et al., 2014). These results require confirmed in clinical trials with a larger patient sample.

These results suggest that *P. mume* may useful as an herbal remedy to treat and prevent some cardiovascular diseases.

### Antitumor Effects

The antitumor effects of *P. mume* have been an important focus of pharmacological studies of the plant in recent years. MK615 and other compounds extracted from *P. mume* have exhibited anti-proliferative activity *in vitro* on many human cancer cell lines (Jeong et al., 2006), for example, the human hepatocellular carcinoma cell lines HuH7, HepG2, and Hep3B (Okada et al., 2007; Sakuraoka et al., 2010); human colon cancer cell lines SW480, COLO, and WiDr (Mori et al., 2007; Cho et al., 2019); human pancreatic cancer cell lines PANC-1, PK-1, PK45H, and MIAPaCa-2 cells (Toshie, 2008; Hattori et al., 2013); human malignant melanoma cell lines SK-MEL28 and A375 cells (Tada et al., 2012); human breast cancer cell lines MDA-MB-468 and MCF-7 cells (Nakagawa et al., 2007); human lung cancer cell lines A549 and PC14 cells (Sunage et al., 2011); and human leukemia cell lines HIMeg, HL-60, and Su9T01 cells (Shen et al., 1995; Kai et al., 2011). The proposed antitumor mechanisms, involved directly suppressing Aurora A and Aurora B kinase activity, inhibition of NF-κB activation (Toshie, 2008), triggering of apoptosis and autophagy (Mori et al., 2007), inducing accumulation of ROS in cancer cells but not in normal endothelial cells (Hattori et al., 2013), inhibition of the ERK1/2 and DNA binding-1 (Id-1) pathways, decreasing Bcl-2 expression (Tada et al., 2012), and suppressing hypoxia tolerance by up-regulation of E-cadherin in cancer cells with mutant KRAS (Nishi et al., 2020).

Anti-neoplastic *in vivo* studies have also shown that MK615 significantly inhibited the growth of human cancer cells in xenograft mice. The effects might be associated with the antioxidant capacity of MK615 (Hattori et al., 2013). Fermented *P. mume* and probiotic treatment also alleviated the 12-dimethylbenz [a]anthracene and 12-O-tetradecanoyl phorbol-13-acetate-induced skin carcinogenesis by mitigating oxidative stress (Lee et al., 2013). In addition, a recent study found that MK615 activated T cell-mediated immunity through programmed death-ligand 1(PD-L1) down-regulation (Yanaki et al., 2018).

When a *P. mume* extract was combined with other anticancer drugs, the drugs showed additive and synergistic effects in different pharmacological models. For example, MK615 enhanced the apoptosis activity of bendamustine in lymphoma cell lines (Inoue et al., 2017). The triterpene extract from *P. mume* augmented the suppressive effects of 5-fluorouracil on esophageal cancer cell xenografts in the peritoneal cavity of a severe combined immunodeficient (SCID) mouse (Yamai et al., 2012). A MK615 and gemcitabine combined treatment was more effective than single treatments in inhibiting the growth of human pancreatic cancer cell xenografts in athymic nude mice (Hattori et al., 2013).

In a clinical setting, Matsushita et al. (2010) described a patient with malignant melanoma who was administered 13 g daily oral doses of MK615 for 5°months, and whose cutaneous in-transit metastatic lesions were significantly reduced, and the apoptotic index of tumor cells significantly increased. In another case report, a hepatocellular carcinoma (HCC)-recurrent patient was administered 6.15 g MK615 twice daily. After 3°months of treatment, the alpha-fetoprotein level decreased, and both the lymph nodes and pulmonary metastases decreased in size (Hoshino et al., 2013). A phase I clinical trial found that patients showed good tolerance to gemcitabine when it was combined with MK615 (Moriyama et al., 2018). A randomized placebo-controlled clinical trial recruited 208 breast cancer patients with diarrhea caused by lapatinib and capecitabine (Xing et al., 2018). The patients were randomized and assigned to two groups given either 100 mg ethanol extract of *P. mume* or placebo, respectively. Diarrhea and gastrointestinal symptoms were assessed using the seven-point Likert scale, two scale forms assessed quality of life of patients, and the SF-36 questionnaire, and Hospital Anxiety and Depression Scale (HADS) were used to evaluate the effects of *P. mume* on diarrhea of those patients. After six°weeks of treatment, the average scores of the Likert scale and HADS were reduced and SF-36 scores were improved significantly in *P. mume* extract treated group when compared to the control group. The results demonstrated that the ethanol extract of *P. mume* relieved diarrhea and gastrointestinal symptoms and improved life quality of breast cancer patients with diarrhea caused by lapatinib and capecitabine. Choi et al. (2002) found that consumption of *P. mume* extracts with a nitrate- and amine-rich diet inhibited endogenous nitrosamine formation in humans, and thus resulted in a lower cancer risk. Overall, numerous studies have shown that *P. mume* possesses antitumor properties and can be used as complimentary therapy for malignant tumors, but the effective constituents and the mechanism of action are worthy of further confirmation.

### Antimicrobial and Antiviral Activity

Several studies have suggested that *P. mume* possesses a wide range of antibacterial activities. Two independent research groups found that *P. mume* extracts inhibited common periodontal bacteria, such as *Porphyromonas gingivalis, Aggregatibacter actinomycetemcomitans*, and cariogenic bacteria such as *Streptococcus mitis, S. sanguis,* and *S. mutans in vitro* (Wong et al., 2010; Seneviratne et al., 2011). It also inhibited bacteria biofilm formation in the mouth cavity (Morimoto-Yamashita et al., 2011). In a six-month randomized single-blinded parallel-controlled clinical trial, a mouthrinse containing *P. mume* showed beneficial effects in patients with fixed orthodontic appliances by decreasing the bleeding index (Chen et al., 2012).

Some studies showed that the herbal combination of *P. mume*, Schizandrae Fructus, and Coptidis Rhizoma inhibited some *Salmonella* and *Escherichia coli* (*E. coli*) strains *in vitro* and *in vivo* (Kwon et al., 2008; Lee and Stein, 2011). *P. mume* also inhibited vero-toxin release from some *E. coli* strains (Sakagami, 2001). Mitani et al. (2018) attributed the antimicrobial activity of *P. mume* on enterobacteria to the phenolic compounds it contains, but not to the free citric acid. Besides its effects on enterobacteria, *P. mume* also inhibited the growth of *Klebsiella pneumoniae* strains, partly by down-regulating the mRNA levels of the capsular polysaccharide (CPS) biosynthesis genes, decreasing CPS production, and reducing bacterial resistance to the host’s immune system (Lin et al., 2013).

In antiviral studies, a fruitjuice concentrate of *P. mume* inhibited human influenza A virus infection before viral adsorption in Mard in Darby canine kidney (MDCK) cells, presumably through activity of a heat-stable lectin-like molecule (Yingsakmongkon et al., 2008). Furan derivatives and phenolics extract might be the active antiviral components of *P. mume* relevant to the inhibition of multiplication of influenza pandemic virus and several other RNA and DNA viruses (Sriwilaijaroen et al., 2011; Ikeda et al., 2019). A recent study showed that the umesu phenolics obtained from *P. mume* inhibited the multiplication of herpes simplex virus (HSV) and might prevent superficial HSV infections (Nishide et al., 2019).

### Immunomodulatory Effects

Jung et al. (2010) found that continuous feeding with fermented *P. mume* and probiotics for four°weeks increased the macrophage ratio in peripheral blood and the T lymphocyte ratio in the spleen in Institute of Cancer Research (ICR)-bred mice. This specific diet also significantly increased antibody production and enhanced the mRNA expression of TNF-α and INF-γ in the splenocytes of experimentally infected mice by killing *Bordetella bronchiseptica*. The immune-enhancing effect of the diet has also been proven in broiler chicks infected with *Salmonella gallinarum* (Jung et al., 2010a). In another study, an ethanol extract of *P. mume* increased the IL-12p40 concentration in the serum and the T cell ratio in the spleen in C57BL/6 J mice (Tsuji et al., 2014). Furthermore, in the tumor-bearing mouse, MK615 treatment enhanced the CD4+/CD8+ ratios following irradiation and reduced tumor volume compared with the irradiated only group (Al-Jahdari et al., 2010). In the field of organ transplantation rejection, Lu et al. (2018) found that an herbal formula containing *P. mume* inhibited both the mammalian target of rapamycin (mTOR) and the NF-κB signaling pathways and significantly inhibited murine skin allograft rejection. The results of all these studies indicated that *P. mume* has dual-directional regulatory effects on mammalian immune system.

### Anti-Inflammatory and Antioxidant Activities

*P. mume* was found to have anti-inflammatory activity in various investigations. Extracellular high-mobility group box-1 protein (HMGB1) is a potent inflammatory agent that can promote the release of pro-inflammatory mediators such as TNF-α. The triterpenoid compounds extracted from *P. mume*, such as oleanolic acid (compound 133), inhibited HMGB1 release from lipopolysaccharide (LPS)-stimulated RAW246.7 cells via the Nrf2/HO-1 pathway (Kawahara et al., 2009). MK615 or a water extract of *P. mume* inhibited the production of cytokines induced by LPS in RAW246.7 cells and in gingival fibroblast cells. The action was mediated by inhibiting the phosphorylation of ERK1/2, p38MAPK, and c-Jun N-terminal kinases (JNK), and blocking LPS-triggered NF-κB activation (Choi H-J. et al., 2007; Morimoto et al., 2009; Morimoto-Yamashita et al., 2015). In the atopic dermatitis animal model, treatment with fermented *P. mume* with probiotics significantly inhibited the development of skin lesions and decreased the peripheral eosinophil ratio and serum concentrations of IgE. In addition, the mRNA expression levels of IL-4, and TNF-α in the spleen were reduced, while the serum concentrations of IL-10 increased (Jung et al., 2010b).

Different radical-scavenging tests, such as the 2,2-diphenyl-1-picrylhydrazyl (DPHH) test and the superoxide anion radical $\left(\cdot {\text{O}}_{2}^{-}\right)$ test, are frequently used methods for determining the antioxidant activities of compounds. Numerous studies have shown that compounds extracted from different parts of the *P. mume* plant exhibited radical-scavenging effect in the DPHH and $\left(\cdot {\text{O}}_{2}^{-}\right)$ tests (Karakaya et al., 2001; Matsuda et al., 2003; Zhang et al., 2015; Pi and Lee, 2017). Hydrogen peroxide (H_2_O_2_) can increase the generation of intracellular ROS leading to DNA damage and apoptosis. An ethanol extract of *P. mume* fruit activated the Nrf2/HO-1 pathway and attenuated H_2_O_2_-induced oxidative stress and apoptosis in the murine skeletal muscle myoblast cell line C2C12 (Kang et al., 2016). Compounds derived from *P. mume* seeds also protected granulose cells from H_2_O_2_- induced apoptosis and promoted estradiol secretion (Kono et al., 2014). In a recent study, Jang et al. (2018) found that a new compound isolated from *P. mume* increased the concentrations of aldehyde dehydrogenase (ALDH) and Werner’s syndrome protein (WRN) in a dose-dependent manner and protected human bronchial epithelial cells and human epidermal keratinocytes from cigarette smoke-induced oxidative damage and DNA damage.

Inflammatory and oxidative stress are the etiology of many diseases. The anti-inflammatory and antioxidant activities of *P. mume* may be the underlying cause of its pharmacological properties. However, most of the antioxidant studies we collected are *in vitro*-based studies. Only a clinical experiment assessed oxidative parameters in the study (Beretta et al., 2016), thus the antioxidative properties of *P. mume* need to be confirmed by more studies *in vivo* in the future.

### Other Pharmacological Activities

Besides the pharmacological effects mentioned above, some scattered studies have reported additional activities induced by *P. mume*. Compounds in the *P. mume* extract, especially acylated quinic acid, inhibited melanogenesis and showed no cytotoxicity in theophylline-stimulated B16 melanoma cells (Nakamura et al., 2013a; Pi and Lee, 2017), indicating that *P. mume* might possess a skin-whitening effect. Interestingly, the effect can be strengthened by fermentation with *Poria cocos* mycelium (Kang et al., 2019). In folk remedies, *P. mume* has been reported to promote salivation. Some formulations, such as San Gan Hua Yin, which contain *P. mume*, can significantly improve the salivary flow rate and mitigate the severity of xerostomia in cancer patients (Murakami et al., 2009). Diets supplemented with *P. mume* extract significantly reduced serum ammonia concentration, elevated hepatic and muscle glycogen concentrations, increased lactate dehydrogenase, citrate synthase, and glutathione peroxidase activities, and decreased creatine kinase activity in skeletal muscles, and as a result, ameliorated exercise-induced fatigue, improved running endurance in rats. The function might relate to enhancing the oxidative capacity of skeletal muscle and inducing the muscle to prefer fatty acids for fuel use rather than amino acids or carbohydrates (Kim et al., 2008; Kim et al., 2020). A cross-sectional pilot study found that patients who receiving *P. mume* regularly had a significantly lower odds ratio (OR) for the presence of allergy symptoms. In the same study, oral treatment with *P. mume* extract attenuated the passive cutaneous anaphylaxis (PCA) reaction and mast cell degranulation in IgE-sensitized mice. The anti-allergic activity might relate to compounds including vanillin, syringic acid, protocatechuic aldehyde, lyoniresinol, and *p*-coumaric acid (Kono et al., 2018). Ina et al. (2002) found that the chlorogenic acid extract from *P. mume* reduced bradykinin and prostaglandin E2 production, inhibited acetic acid-induced writhing behavior in mice, and showed analgesic effects. The compound also relieved the tension caused by ether stresses in menopausal model rats, because it recovered catecholamine levels and decreased the adrenocorticotropic hormone (ACTH) levels in the plasma of model rats (Ina et al., 2004).

### Toxicity and Safety

A systematic toxicology study evaluated the safety of the ethanol extract of *P. mume*. The oral acute test showed no lethal effects in rats and mice at the maximum tolerated dose of 20 g/kg. In the subacute toxicity test, no adverse effects were observed at doses greater than 3.33 g/kg body weight for 30°days. In addition, no mutagenic or genotoxic effects were observed in the experiments, including the Ames test, the micronucleus test, and the sperm abnormality test (Lu et al., 2009). The safety profile of mumefural, a bioactive compound derived from the heated fruit of *P. mume*, was also investigated by acute and subacute oral toxicity experiments. The results indicated that the approximate lethal dose of mumefural in ICR mice was >5 g/kg (Kim et al., 2020).

On the other hand, a few clinical studies evaluated the safety of P. mume during the experiments, and no adverse events were observed (Hoshino et al., 2013; Takemura et al., 2014). These studies demonstrated that *P. mume* could be used safely as a dietary supplement.

However, in recent years, several studies have found that *P. mume* peamaclein (also known as gibberellin-regulated protein or GRP) is a cross-reactive allergen between *P. mume* and peach (*P. persica*), and could cause food-dependent exercise-induced anaphylaxis (Iijima et al., 2015; Inomata et al., 2016; Yamanaka et al., 2019). It might be necessary to remind individuals who are allergic to peaches to avoid eating *P. mume*.

## Conclusions and Future Perspectives

As an important medicinal herb and food commodity, the Japanese apricot or Chinese plum (*P. mume*) has aroused the interest of numerous researchers. In this review, we conducted an exhaustive search of the literature describing the phytochemical and pharmacological properties of *P. mume*. We found that 192 compounds have been isolated from different parts of the plant, including phenolics, organic acids, steroids, terpenes, benzyl glycosides, cyanogenic glycosides, furfurals, lignans, alkaloid, amino acids, and some other compounds. Numerous studies disclosed the pharmacological activities of *P. mume*, including its anti-diabetic, antihyperlipidemic, lowering uric acid, anti-osteoporosis, hepatoprotection, anti-*H. pylori*, stimulating intestinal motility, anti-inflammatory, antioxidant, improving blood fluidity effects, as well as its inhibiting platelet aggregation, anti-tumor, antimicrobial, antiviral, immunomodulation, skin whitening, stimulating salivary secretion, anti-fatigue, anti-allergic, and analgesic properties.

Several studies have established connections between the chemical compositions and the pharmacological properties of the plant. For example, the phenolic compounds confer its antidiabetic (Lee et al., 2016), antimicrobial (Mitani et al., 2018), antiviral (Ikeda et al., 2019), and anti-oxidative (Xia et al., 2010) activities; organic acid components exert hypolipidemic (Choi et al., 2007) and antibacterial (Gao, 2012) effects; and steroids and terpenes inhibit osteoclast differentiation (Yan et al., 2015). However, most of the pharmacological studies of *P. mume* are based on crude extracts, refined preparations such as MK615, and formulas containing *P. mume* such as *wu mei wan* or TCM decoctions. Thus, studies elucidating the relationships between the pharmacodynamics and the bioactive constituents of the plant still require further investigation.

Among the pharmacological properties of *P. mume,* the antidiabetic (Tu et al., 2013) and hepatoprotective effects (Hokari, 2012; Beretta et al., 2016), and the inhibitory effects on chronic gastritis (Enomoto et al., 2010) and gastroesophageal reflux (Maekita, 2015), blood pressure lowering effects (Takemura et al., 2014), and antitumor activities (Matsushita et al., 2010; Hoshino et al., 2013)are particularly notable. Because these pharmacological properties have been proven not only by *in vitro* and *in vivo* experiments but also by several clinical studies. However, most clinical studies have only involved small-sample clinical trials or case reports. Thus, to provide strong evidence of clinical applications, well-designed randomized controlled trials, cohort studies, nested case-control studies, and real-world studies need to be carried out appropriately in the future.

With regard to the safety profile of *P. mume*, existing studies have provided only limited information. More systematic toxicology studies still need to be carried out in the future on the aqueous extraction of *P. mume*, refined products such as MK 615, and pharmacodynamic components of the plant. The side-effect associating to the *P. mume* usage observed in the clinical experiments also needs to be identified and reported in future clinical studies.

In terms of quality control, the information about the harvest season and the maturity level of the fruit, the quantitative studies of the index components are scarcely in the existing studies. These should be emphasized in the future to promote the reproducibility of the studies.

In summary, this review provided a comprehensive information regarding *P. mume*, raised limitations of existing studies, and proposed future research directions, and has established a groundwork for further utilization and development of the plant.
